# 2-Hy­droxy-3-oct­yloxy-*N*,*N*,*N*-trimethyl­propan-1-aminium bromide

**DOI:** 10.1107/S1600536810040705

**Published:** 2010-10-20

**Authors:** Jiuqiang Liu, Zengbin Wei, Xilian Wei, Chong Zhang

**Affiliations:** a College of Chemistry and Chemical Engineering, Liaocheng University, Shandong 252059, People’s Republic of China

## Abstract

In the title compound, C_14_H_32_NO_2_^+^·Br^−^, organic cationsstacked parallel to the *a* axis andbromide anions placed between the head groups of the cations form ionic pairs *via* weak inter­molecular O—H⋯Br hydrogen bonds. The octyl chain in the cation adopts an all-*trans* conformation. The O—CH_2_—CH(—OH)—CH_2_ portion of the molecule is disordered over two sets of sites with occupancy factors of 0.57 (3) and 0.47 (3).

## Related literature

For uses of cationic surfacta­nts, see: Zhao *et al.* (1997 ▶, 2010 ▶). For bond lengths and angles, see: Koh *et al.* (1993 ▶). 
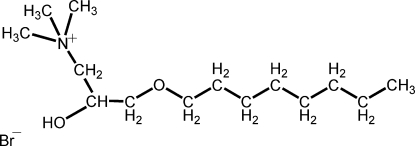


## Experimental

### 

#### Crystal data

C_14_H_32_NO_2_^+^·Br^−^
*M* *_r_* = 326.32
Monoclinic, 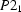

*a* = 5.9713 (11) Å
*b* = 7.4780 (12) Å
*c* = 19.992 (2) Å
β = 92.923 (1)°
*V* = 891.6 (2) Å^3^
*Z* = 2
Mo *K*α radiation
μ = 2.30 mm^−1^
*T* = 298 K
0.42 × 0.30 × 0.04 mm

#### Data collection

Siemens SMART CCD area-detector diffractometer
Absorption correction: multi-scan (*SADABS*; Sheldrick, 1996 ▶) *T*_min_ = 0.445, *T*_max_ = 0.914
4642 measured reflections
2827 independent reflections
1168 reflections with *I* > 2σ(*I*)
*R*_int_ = 0.135

#### Refinement

*R*[*F* ^2^ > 2σ(*F* ^2^)] = 0.081
*wR*(*F* ^2^) = 0.199
*S* = 1.03
2827 reflections
211 parameters
1 restraint
H-atom parameters constrained
Δρ_max_ = 0.74 e Å^−3^
Δρ_min_ = −0.30 e Å^−3^
Absolute structure: Flack (1983 ▶), **1124 Friedel pairs**
Flack parameter: 0.02 (7)

### 

Data collection: *SMART* (Siemens, 1996 ▶); cell refinement: *SAINT* (Siemens, 1996 ▶); data reduction: *SAINT*; program(s) used to solve structure: *SHELXS97* (Sheldrick, 2008 ▶); program(s) used to refine structure: *SHELXL97* (Sheldrick, 2008 ▶); molecular graphics: *SHELXTL* (Sheldrick, 2008 ▶); software used to prepare material for publication: *SHELXTL*.

## Supplementary Material

Crystal structure: contains datablocks I, global. DOI: 10.1107/S1600536810040705/jj2054sup1.cif

Structure factors: contains datablocks I. DOI: 10.1107/S1600536810040705/jj2054Isup2.hkl

Additional supplementary materials:  crystallographic information; 3D view; checkCIF report

## Figures and Tables

**Table 1:** Hydrogen-bond geometry (Å, °)

*D*—H⋯*A*	*D*—H	H⋯*A*	*D*⋯*A*	*D*—H⋯*A*
O2—H2⋯Br1^i^	0.82	2.50	3.32 (3)	171
O2′—H2′⋯Br1^ii^	0.82	2.27	3.05 (4)	160

Symmetry codes: (i) 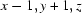; (ii) 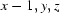.

**Fig. 1.**
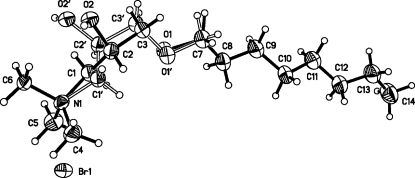
The molecular structure of the title compound, with atom labels and 50% probability displacement ellipsoids. Atoms C1:C1', C2:C2', C3:C3', O1:O1' and O2:O2' with disordered site occupancies 0.47 (3):0.53 (3) are shown.

**Fig. 2.**
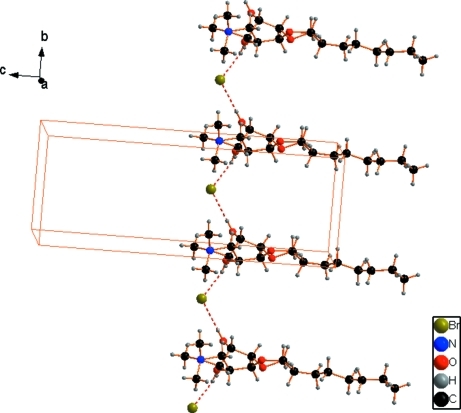
Crystal packing of the title compound, showing one extended chain structure, linked by weak O—H···Br hydrogen bonds (dashed lines).

## supplementary crystallographic information

### Comment

Cationic surfactants have attracted much attention due to their wide spread use in both household and industrial activities, such as in the production of cosmetics (Zhao *et al.*, 1997) and polluted soil treatment (Zhao, *et al.*, 2010). As a contribution to the chemistry of surfactants, we report here the synthesis and crystal structure of the title compound, C_14_H_32_Br_1_N_1_O_2_.

The asymmetric unit of the title compound consists of a 3-octyloxy-2-hydroxypropyl-*N*,*N*,*N*- trimethylpropan-1-aminium cation, and a bromide anion, (Fig. 1). Atoms C1:C1', C2:C2', C3:C3', O1:O1' and O2:O2' are disordered with site occupancies of 0.47 (3):0.53 (3). The C—C bond distances in the octyl chain are alternately short and long, the average of the short distances being 1.46 (6)Å and the average of the long distances being 1.49 (8) Å. All N—C bond lengths and C—N—C angles are within the usual ranges (Koh *et al.*, 1993). The bond distances of O1—C3 and O1—C7 are 1.4 (3)and 1.44 (18) Å, respectively. The octyl chains of the cations form monolayers parallel to the (010) plane. Adjacent anions are connected by weak intermolecular O—H···Br interactions and organic cations stacked parallel along the *a* axis (Table 1, Fig. 2).

### Experimental

The reaction was carried out under nitrogen atmosphere. Trimethylammonium bromide (0.12 mol) and octyl glycidyl ether (0.1 mol) were added to a stirred solution of ethanol (100 ml) and stirred at 315 K for 24 h. The resulting clear solution was evaporated under vacuum. Colourless crystals suitable for X-ray analysis were obtained by slow evaporation of an ethyl acetate solution over a period of two weeks. (yield 82%, m.p.340k) Anal. Calcd (%) for C_14_H_32_Br_1_N_1_O_2_ (Mr = 326.32): C, 51.48; H, 9.81; N, 4.29. Found (%): C, 51.52; H, 9.83; N, 4.26.

### Refinement

All H atoms were placed geometrically and treated as riding on their parent atoms with O—H = 0.82 Å, C—H = 0.97 (methylene) Å [*U*_iso_(H) = 1.2*U*_eq_(C)], and C—H = 0.96 (methyl) Å [*U*_iso_(H) = 1.5*U*_eq_(C)]. Atoms C1, C2, C3, O1 and O2 were found to be disordered over two sites, and the ratio of the occupancy factors refined to 0.47 (3):0.53 (3), 0.47 (3):0.53 (3), 0.47 (3):0.53 (3), 0.47 (3):0.53 (3) and 0.47 (3):0.53 (3), for atoms C1:C1', C2:C2', C3:C3', O1:O1' and O2:O2', respectively.

### Crystal data

**Table:**

C_14_H_32_NO_2_^+^·Br^−^	*F*(000) = 348
*M**_r_* = 326.32	*D*_x_ = 1.216 Mg m^−^^3^
Monoclinic, *P*2_1_	Mo *K*α radiation, λ = 0.71073 Å
Hall symbol: P 2yb	Cell parameters from 889 reflections
*a* = 5.9713 (11) Å	θ = 3.1–28.4°
*b* = 7.4780 (12) Å	µ = 2.30 mm^−^^1^
*c* = 19.992 (2) Å	*T* = 298 K
β = 92.923 (1)°	Block, colourless
*V* = 891.6 (2) Å^3^	0.42 × 0.30 × 0.04 mm
*Z* = 2	

### Data collection

**Table:**

Siemens SMART CCD area-detector diffractometer	2827 independent reflections
Radiation source: fine-focus sealed tube	1168 reflections with *I* > 2σ(*I*)
graphite	*R*_int_ = 0.135
phi and ω scans	θ_max_ = 25.0°, θ_min_ = 2.0°
Absorption correction: multi-scan (SABABS; Sheldrick, 1996)	*h* = −7→7
*T*_min_ = 0.445, *T*_max_ = 0.914	*k* = −7→8
4642 measured reflections	*l* = −19→23

### Refinement

**Table:**

Refinement on *F*^2^	Secondary atom site location: difference Fourier map
Least-squares matrix: full	Hydrogen site location: inferred from neighbouring sites
*R*[*F*^2^ > 2σ(*F*^2^)] = 0.081	H-atom parameters constrained
*wR*(*F*^2^) = 0.199	*w* = 1/[σ^2^(*F*_o_^2^) + (0.0713*P*)^2^] where *P* = (*F*_o_^2^ + 2*F*_c_^2^)/3
*S* = 1.03	(Δ/σ)_max_ = 0.001
2827 reflections	Δρ_max_ = 0.74 e Å^−^^3^
211 parameters	Δρ_min_ = −0.30 e Å^−^^3^
1 restraint	Absolute structure: Flack (1983), 1124 FRIEDEL PAIRS
Primary atom site location: structure-invariant direct methods	Flack parameter: 0.02 (7)

### Special details

**Table:**

Geometry. All e.s.d.'s (except the e.s.d. in the dihedral angle between two l.s. planes) are estimated using the full covariance matrix. The cell e.s.d.'s are taken into account individually in the estimation of e.s.d.'s in distances, angles and torsion angles; correlations between e.s.d.'s in cell parameters are only used when they are defined by crystal symmetry. An approximate (isotropic) treatment of cell e.s.d.'s is used for estimating e.s.d.'s involving l.s. planes.
Refinement. Refinement of *F*^2^ against ALL reflections. The weighted *R*-factor *wR* and goodness of fit *S* are based on *F*^2^, conventional *R*-factors *R* are based on *F*, with *F* set to zero for negative *F*^2^. The threshold expression of *F*^2^ > σ(*F*^2^) is used only for calculating *R*-factors(gt) *etc*. and is not relevant to the choice of reflections for refinement. *R*-factors based on *F*^2^ are statistically about twice as large as those based on *F*, and *R*- factors based on ALL data will be even larger.

### Fractional atomic coordinates and isotropic or equivalent isotropic displacement parameters (Å^2^)

**Table:**

	*x*	*y*	*z*	*U*_iso_*/*U*_eq_	Occ. (<1)
Br1	0.66822 (19)	0.4416 (9)	0.40071 (6)	0.0909 (6)	
N1	0.0535 (12)	0.941 (5)	0.3976 (4)	0.070 (2)	
O1	−0.02 (4)	0.902 (10)	0.186 (9)	0.09 (2)	0.57 (3)
O2	−0.389 (6)	1.064 (4)	0.3144 (15)	0.077 (10)	0.57 (3)
H2	−0.3915	1.1580	0.3356	0.116*	0.57 (3)
C1	−0.039 (17)	0.883 (12)	0.330 (5)	0.08 (2)	0.57 (3)
H1A	−0.1382	0.7816	0.3346	0.099*	0.57 (3)
H1B	0.0832	0.8449	0.3028	0.099*	0.57 (3)
C2	−0.168 (12)	1.034 (9)	0.293 (3)	0.079 (16)	0.57 (3)
H2A	−0.0811	1.1452	0.2950	0.094*	0.57 (3)
C3	−0.205 (15)	0.969 (10)	0.221 (4)	0.08 (3)	0.57 (3)
H3A	−0.3176	0.8756	0.2211	0.100*	0.57 (3)
H3B	−0.2699	1.0678	0.1952	0.100*	0.57 (3)
O1'	−0.01 (5)	0.970 (11)	0.192 (12)	0.09 (4)	0.43 (3)
O2'	−0.364 (7)	0.801 (5)	0.3282 (19)	0.077 (13)	0.43 (3)
H2'	−0.3683	0.6949	0.3386	0.116*	0.43 (3)
C1'	−0.02 (2)	0.979 (13)	0.326 (6)	0.08 (3)	0.43 (3)
H1'1	0.1120	0.9954	0.3008	0.097*	0.43 (3)
H1'2	−0.1025	1.0912	0.3248	0.097*	0.43 (3)
C2'	−0.168 (16)	0.834 (12)	0.292 (4)	0.08 (2)	0.43 (3)
H2'1	−0.0803	0.7237	0.2939	0.095*	0.43 (3)
C3'	−0.22 (2)	0.870 (17)	0.218 (6)	0.09 (3)	0.43 (3)
H3'1	−0.3563	0.9451	0.2117	0.104*	0.43 (3)
H3'2	−0.2504	0.7592	0.1934	0.104*	0.43 (3)
C4	0.190 (5)	1.104 (5)	0.4099 (15)	0.099 (11)	
H4A	0.2923	1.1176	0.3747	0.148*	
H4B	0.2727	1.0933	0.4521	0.148*	
H4C	0.0932	1.2064	0.4107	0.148*	
C5	0.206 (4)	0.784 (5)	0.4109 (15)	0.096 (10)	
H5A	0.3187	0.7822	0.3783	0.144*	
H5B	0.1202	0.6759	0.4079	0.144*	
H5C	0.2766	0.7947	0.4549	0.144*	
C6	−0.1293 (12)	0.936 (4)	0.4460 (4)	0.078 (3)	
H6A	−0.0659	0.9507	0.4907	0.116*	
H6B	−0.2045	0.8222	0.4425	0.116*	
H6C	−0.2347	1.0298	0.4358	0.116*	
C7	−0.0337 (12)	0.978 (4)	0.1194 (4)	0.104 (8)	
H7A	−0.0355	1.1079	0.1219	0.125*	0.57 (3)
H7B	−0.1696	0.9387	0.0952	0.125*	0.57 (3)
H7C	−0.0705	1.0997	0.1058	0.125*	0.43 (3)
H7D	−0.1588	0.9023	0.1052	0.125*	0.43 (3)
C8	0.169 (2)	0.915 (5)	0.0850 (6)	0.103 (7)	
H8A	0.2919	0.9934	0.0992	0.123*	
H8B	0.2056	0.7973	0.1025	0.123*	
C9	0.168 (2)	0.903 (4)	0.0123 (6)	0.112 (9)	
H9A	0.0434	0.9736	−0.0059	0.135*	
H9B	0.1380	0.7795	0.0001	0.135*	
C10	0.375 (2)	0.961 (5)	−0.0225 (6)	0.109 (5)	
H10A	0.5057	0.9083	0.0006	0.131*	
H10B	0.3888	1.0895	−0.0193	0.131*	
C11	0.371 (3)	0.907 (5)	−0.0954 (6)	0.122 (10)	
H11A	0.3381	0.7804	−0.0983	0.146*	
H11B	0.2489	0.9700	−0.1188	0.146*	
C12	0.581 (2)	0.941 (7)	−0.1320 (6)	0.117 (4)	
H12A	0.6494	1.0475	−0.1121	0.141*	
H12B	0.6818	0.8427	−0.1210	0.141*	
C13	0.581 (3)	0.964 (7)	−0.2030 (7)	0.131 (8)	
H13A	0.5496	1.0895	−0.2123	0.157*	
H13B	0.4562	0.8963	−0.2226	0.157*	
C14	0.781 (3)	0.916 (7)	−0.2394 (8)	0.151 (9)	
H14A	0.9120	0.9673	−0.2171	0.227*	
H14B	0.7654	0.9609	−0.2843	0.227*	
H14C	0.7959	0.7882	−0.2404	0.227*	

### Atomic displacement parameters (Å^2^)

**Table:**

	*U*^11^	*U*^22^	*U*^33^	*U*^12^	*U*^13^	*U*^23^
Br1	0.0819 (8)	0.0684 (8)	0.1204 (10)	−0.001 (2)	−0.0165 (6)	0.005 (2)
N1	0.056 (4)	0.076 (6)	0.078 (6)	0.00 (2)	0.007 (5)	0.011 (19)
O1	0.10 (4)	0.10 (3)	0.08 (4)	0.00 (6)	0.01 (3)	0.01 (5)
O2	0.067 (18)	0.078 (16)	0.087 (17)	−0.005 (12)	0.003 (12)	−0.006 (12)
C1	0.08 (4)	0.09 (3)	0.07 (5)	0.00 (5)	0.01 (3)	0.01 (5)
C2	0.08 (4)	0.08 (4)	0.08 (4)	0.00 (3)	0.01 (4)	0.01 (3)
C3	0.08 (4)	0.09 (8)	0.07 (4)	0.00 (4)	0.00 (3)	0.01 (4)
O1'	0.10 (6)	0.10 (8)	0.08 (5)	0.00 (8)	0.01 (4)	0.01 (7)
O2'	0.07 (2)	0.08 (2)	0.09 (2)	−0.005 (15)	0.003 (17)	−0.006 (16)
C1'	0.08 (5)	0.09 (8)	0.07 (6)	0.00 (6)	0.01 (4)	0.01 (6)
C2'	0.08 (5)	0.09 (6)	0.08 (6)	0.00 (4)	0.01 (5)	0.01 (4)
C3'	0.09 (5)	0.10 (6)	0.08 (5)	0.00 (6)	0.01 (4)	0.01 (5)
C4	0.09 (2)	0.10 (3)	0.11 (3)	−0.015 (18)	0.00 (2)	0.022 (18)
C5	0.08 (2)	0.09 (3)	0.12 (3)	0.026 (17)	0.010 (19)	−0.003 (17)
C6	0.060 (6)	0.103 (9)	0.070 (6)	0.007 (19)	0.008 (5)	0.020 (19)
C7	0.105 (10)	0.12 (2)	0.084 (10)	−0.003 (12)	0.005 (8)	0.007 (13)
C8	0.108 (10)	0.12 (2)	0.078 (9)	0.000 (14)	0.008 (7)	0.011 (14)
C9	0.119 (11)	0.14 (3)	0.080 (10)	0.000 (13)	0.000 (8)	0.006 (12)
C10	0.124 (10)	0.128 (15)	0.074 (9)	−0.01 (2)	−0.001 (7)	0.016 (17)
C11	0.127 (12)	0.15 (3)	0.084 (10)	−0.008 (15)	0.000 (9)	0.004 (13)
C12	0.133 (11)	0.134 (12)	0.086 (10)	0.00 (4)	0.003 (8)	0.01 (3)
C13	0.139 (13)	0.16 (2)	0.090 (11)	0.00 (2)	0.005 (9)	0.01 (2)
C14	0.163 (15)	0.19 (3)	0.098 (11)	0.03 (3)	0.009 (11)	0.01 (2)

### Geometric parameters (Å, °)

**Table:**

N1—C4	1.48 (4)	C5—H5C	0.9600
N1—C6	1.495 (10)	C6—H6A	0.9600
N1—C1'	1.50 (14)	C6—H6B	0.9600
N1—C5	1.50 (4)	C6—H6C	0.9600
N1—C1	1.51 (11)	C7—C8	1.50 (2)
O1—C3	1.4 (2)	C7—H7A	0.9700
O1—C7	1.44 (17)	C7—H7B	0.9700
O2—C2	1.42 (6)	C7—H7C	0.9703
O2—H2	0.8200	C7—H7D	0.9698
C1—C2	1.53 (12)	C8—C9	1.456 (16)
C1—H1A	0.9700	C8—H8A	0.9700
C1—H1B	0.9700	C8—H8B	0.9700
C2—C3	1.53 (10)	C9—C10	1.51 (2)
C2—H2A	0.9800	C9—H9A	0.9700
C3—H3A	0.9700	C9—H9B	0.9700
C3—H3B	0.9700	C10—C11	1.51 (2)
O1'—C3'	1.6 (3)	C10—H10A	0.9700
O2'—C2'	1.43 (7)	C10—H10B	0.9700
O2'—H2'	0.8200	C11—C12	1.501 (19)
C1'—C2'	1.54 (16)	C11—H11A	0.9700
C1'—H1'1	0.9700	C11—H11B	0.9700
C1'—H1'2	0.9700	C12—C13	1.431 (18)
C2'—C3'	1.53 (13)	C12—H12A	0.9700
C2'—H2'1	0.9800	C12—H12B	0.9700
C3'—H3'1	0.9700	C13—C14	1.47 (3)
C3'—H3'2	0.9700	C13—H13A	0.9700
C4—H4A	0.9600	C13—H13B	0.9700
C4—H4B	0.9600	C14—H14A	0.9600
C4—H4C	0.9600	C14—H14B	0.9600
C5—H5A	0.9600	C14—H14C	0.9600
C5—H5B	0.9600		
			
C4—N1—C6	109 (3)	H6A—C6—H6C	109.5
C4—N1—C1'	98 (5)	H6B—C6—H6C	109.5
C6—N1—C1'	116 (5)	O1'—C7—O1	21 (5)
C4—N1—C5	106.9 (8)	O1'—C7—C8	114 (10)
C6—N1—C5	109 (2)	O1—C7—C8	107 (8)
C1'—N1—C5	118 (5)	O1'—C7—H7A	89.5
C4—N1—C1	124 (4)	O1—C7—H7A	110.4
C6—N1—C1	109 (4)	C8—C7—H7A	110.4
C1'—N1—C1	28 (3)	O1'—C7—H7B	121.5
C5—N1—C1	97 (4)	O1—C7—H7B	110.4
C3—O1—C7	108 (10)	C8—C7—H7B	110.4
C2—O2—H2	109.5	H7A—C7—H7B	108.6
N1—C1—C2	112 (7)	O1'—C7—H7C	109.2
N1—C1—H1A	109.3	O1—C7—H7C	129.1
C2—C1—H1A	109.3	C8—C7—H7C	110.0
N1—C1—H1B	109.3	H7A—C7—H7C	22.6
C2—C1—H1B	109.3	H7B—C7—H7C	88.5
H1A—C1—H1B	108.0	O1'—C7—H7D	107.7
O2—C2—C3	104 (6)	O1—C7—H7D	92.8
O2—C2—C1	115 (5)	C8—C7—H7D	108.0
C3—C2—C1	105 (6)	H7A—C7—H7D	126.2
O2—C2—H2A	110.7	H7B—C7—H7D	20.3
C3—C2—H2A	110.7	H7C—C7—H7D	107.8
C1—C2—H2A	110.8	C9—C8—C7	121.2 (14)
O1—C3—C2	120 (9)	C9—C8—H8A	107.0
O1—C3—H3A	107.4	C7—C8—H8A	107.0
C2—C3—H3A	107.4	C9—C8—H8B	107.0
O1—C3—H3B	107.4	C7—C8—H8B	107.0
C2—C3—H3B	107.4	H8A—C8—H8B	106.8
H3A—C3—H3B	106.9	C8—C9—C10	118.8 (16)
C7—O1'—C3'	108 (10)	C8—C9—H9A	107.6
C2'—O2'—H2'	109.5	C10—C9—H9A	107.6
N1—C1'—C2'	115 (8)	C8—C9—H9B	107.6
N1—C1'—H1'1	108.4	C10—C9—H9B	107.6
C2'—C1'—H1'1	108.4	H9A—C9—H9B	107.0
N1—C1'—H1'2	108.4	C9—C10—C11	113.5 (19)
C2'—C1'—H1'2	108.4	C9—C10—H10A	108.9
H1'1—C1'—H1'2	107.5	C11—C10—H10A	108.9
O2'—C2'—C3'	113 (8)	C9—C10—H10B	108.9
O2'—C2'—C1'	111 (7)	C11—C10—H10B	108.9
C3'—C2'—C1'	114 (8)	H10A—C10—H10B	107.7
O2'—C2'—H2'1	106.3	C12—C11—C10	117.1 (18)
C3'—C2'—H2'1	106.3	C12—C11—H11A	108.0
C1'—C2'—H2'1	106.3	C10—C11—H11A	108.0
C2'—C3'—O1'	105 (10)	C12—C11—H11B	108.0
C2'—C3'—H3'1	110.7	C10—C11—H11B	108.0
O1'—C3'—H3'1	110.7	H11A—C11—H11B	107.3
C2'—C3'—H3'2	110.7	C13—C12—C11	123.2 (13)
O1'—C3'—H3'2	110.7	C13—C12—H12A	106.5
H3'1—C3'—H3'2	108.8	C11—C12—H12A	106.5
N1—C4—H4A	109.5	C13—C12—H12B	106.5
N1—C4—H4B	109.5	C11—C12—H12B	106.5
H4A—C4—H4B	109.5	H12A—C12—H12B	106.5
N1—C4—H4C	109.5	C12—C13—C14	120 (2)
H4A—C4—H4C	109.5	C12—C13—H13A	107.3
H4B—C4—H4C	109.5	C14—C13—H13A	107.3
N1—C5—H5A	109.5	C12—C13—H13B	107.3
N1—C5—H5B	109.5	C14—C13—H13B	107.3
H5A—C5—H5B	109.5	H13A—C13—H13B	106.9
N1—C5—H5C	109.5	C13—C14—H14A	109.5
H5A—C5—H5C	109.5	C13—C14—H14B	109.5
H5B—C5—H5C	109.5	H14A—C14—H14B	109.5
N1—C6—H6A	109.5	C13—C14—H14C	109.5
N1—C6—H6B	109.5	H14A—C14—H14C	109.5
H6A—C6—H6B	109.5	H14B—C14—H14C	109.5
N1—C6—H6C	109.5		
			
C4—N1—C1—C2	−52 (8)	N1—C1'—C2'—C3'	−175 (8)
C6—N1—C1—C2	80 (7)	O2'—C2'—C3'—O1'	160 (9)
C1'—N1—C1—C2	−29 (12)	C1'—C2'—C3'—O1'	33 (14)
C5—N1—C1—C2	−168 (6)	C7—O1'—C3'—C2'	169 (9)
N1—C1—C2—O2	−78 (9)	C3'—O1'—C7—C8	−130 (11)
N1—C1—C2—C3	168 (6)	C3—O1—C7—C8	177 (7)
C7—O1—C3—C2	−136 (8)	O1'—C7—C8—C9	176 (6)
O2—C2—C3—O1	−172 (8)	O1—C7—C8—C9	155 (6)
C1—C2—C3—O1	−50 (11)	C7—C8—C9—C10	140 (3)
C4—N1—C1'—C2'	179 (8)	C8—C9—C10—C11	168 (3)
C6—N1—C1'—C2'	−66 (10)	C9—C10—C11—C12	−173 (3)
C5—N1—C1'—C2'	65 (10)	C10—C11—C12—C13	−155 (4)
C1—N1—C1'—C2'	17 (11)	C11—C12—C13—C14	−152 (4)
N1—C1'—C2'—O2'	57 (12)		

### Hydrogen-bond geometry (Å, °)

**Table:**

*D*—H···*A*	*D*—H	H···*A*	*D*···*A*	*D*—H···*A*
O2—H2···Br1^i^	0.82	2.50	3.32 (3)	171
O2'—H2'···Br1^ii^	0.82	2.27	3.05 (4)	160

Symmetry codes: (i) *x*−1, *y*+1, *z*; (ii) *x*−1, *y*, *z*.
